# Dose assessment in dental cone-beam computed tomography: Comparison of optically stimulated luminescence dosimetry with Monte Carlo method

**DOI:** 10.1371/journal.pone.0219103

**Published:** 2020-03-31

**Authors:** Chena Lee, Jeongmin Yoon, Sang-Sun Han, Ji Yeon Na, Jeong-Hee Lee, Young Hyun Kim, Jae Joon Hwang

**Affiliations:** 1 Department of Oral and Maxillofacial Radiology, Yonsei University College of Dentistry, Seoul, Republic of Korea; 2 Department of Radiation Oncology, Seoul National University Hospital, Seoul, Republic of Korea; 3 Department of Radiation Oncology, Sheikh Khalifa Specialty Hospital, Ras Al Khaimah, United Arab Emirates; 4 Department of Oral and Maxillofacial Radiology, School of Dentistry, Pusan National University, Yangsan, Korea; National Taiwan University, School of Dentistry, TAIWAN

## Abstract

The variety of cone-beam computed tomography (CBCT) machines and their applications has rapidly increased in recent years, making the dose evaluation of individual devices an important issue. Patient doses from CBCT were assessed with two different methods: optically stimulated luminescence dosimeter (OSLD) measurements and Monte Carlo (MC) simulations, in four different examination modes. Based on an analysis of the measurement process and the obtained values, a recommendation is made regarding which method is more practical and efficient for acquiring the effective dose of CBCT. Twenty-two OSLDs were calibrated and equipped in human phantoms of head and neck organs. They were exposed to radiation from two CBCT units—CS9300 (Carestream Dental LLC, Atlanta, Georgia) and RAYSCAN α+ (Ray Co. Ltd, Hwaseong-si, Korea)—using two different examination modes. The dose recorded using the OSLDs was used to calculate the organ dose and the effective dose for each unit in each examination mode. These values were also calculated using MC software, PCXMC (STUK, Helsinki, Finland). The organ doses and effective doses obtained using both methods were compared for each examination mode of the individual units. The OSLD-measured effective dose value was higher than that obtained using the MC method for each examination mode, except the dual jaw mode of CS9300. The percent difference of the effective dose between the two methods ranged from 4.0% to 14.3%. The dose difference between the methods decreased as the field of view became smaller. The organ dose values varied according to the method, although the overall trend was similar for both methods. The organs showing high doses were mostly consistent for both methods. In this study, the effective dose obtained by OSLD measurements and MC simulations were compared, and both methods were described in detail. As a relatively efficient and easy-to-perform method, we cautiously suggest using MC simulations for dose evaluations in the future.

## Introduction

The radiation doses of dental diagnostic examinations are relatively low compared to those of medical examinations [1, 2]. However, as cone-beam computed tomography (CBCT) has become widely performed for various purposes in dental clinics, it is no longer valid simply to state that the radiation dose in dentistry is very low.

Although patients’ overall radiation dose in dentistry has increased, dose evaluation methods have not kept pace with these changes. Thermoluminescent dosimetry (TLD) is the traditional method of dose measurement, and most studies of dental radiation doses were based on this method [3]. A recent trend has emerged for TLD to be replaced by optically stimulated luminescence dosimetry (OSLD) or a metal oxide semiconductor field effect transistor (MOSFET) [4, 5]. MOSFET provides a fast reading of the dosage, as it connects directly to an electronic probe. It has generally been considered acceptable for dosimetry in radiotherapy, due to its suitability for high dose ranges [6].

The basic phenomenological fundamentals of OSLD and TLD are the same; TLD measures the energy that was stored during irradiation as it is released in the form of heat, while OSLD does so in the form of light [7]. OSLD has several advantages over TLD, such as high sensitivity, precision, and simple dosimeter preparation and readout [7]. Based on these considerations, a few studies have performed dose measurements with OSLD and reported that it showed reliable results in comparison to the TLD method [8]. Nonetheless, TLD has been a common method of dosimetry in the dental field for a long time, and not many studies have yet investigated OSLD measurements.

The Monte Carlo (MC) method is another dose assessment method, which simulates X-ray photon interactions with body organs and calculates the overall effective dose. This method simulates virtual photon interactions in a human phantom and the expected radiation dose. It has the advantage of being simple to use, since calibration and readout procedures are not required and the result is not dependent on the type of the dosimeter or its location in the phantom [9]. Nonetheless, it is challenging and complex to build precise code for MC calculations to simulate radiation exposures that are exactly the same as those encountered in actual clinical conditions. Accurate simulations must be based on the correct geometry of the machine and radiation beam, including parameters such as the distance from the X-ray source to the patient, the beam rotation angle, and the vertical angle of the X-ray beam. An incorrect combination of those factors could yield a difference in the effective dose of up to 51.24% compared to the TLD-measured value [10].

Both OSLD measurements and the MC simulation method are relatively new techniques in dental X-ray equipment at the present [10, 11]. More research on these newly introduced methods (OSLD or MC calculations) compared to traditional dosimetry should be performed to prove the efficiency of these methods. However, as far as the authors know, no English-language studies have been reported with a comparison of the MC and OSLD methods for dose assessment in the dental field.

In this study, the patient dose from CBCT was assessed with two different methods—OSLD measurements and MC simulations—in two different CBCT units with different examination modes. Based on an analysis of the measurement process and the obtained values, a recommendation is made regarding which method is more practical and efficient for acquiring the effective dose of CBCT.

## Material and methods

### 1. CBCT machines and examination protocols

The CBCT machines used were a CS9300 (Carestream Dental LLC, Atlanta, Georgia) and a RAYSCAN α+ CBCT scanner (Ray Co. Ltd, Hwaseong-si, Korea).

The examination modes of the individual units used in this study were as follows: for the CS9300, facial mode (field of view [FOV] = 17 ×13.5 cm) and dual jaw mode (FOV = 10 × 10 cm); for the RAYSCAN α+, large jaw mode (FOV = 16 × 10 cm) and jaw mode (FOV = 10 × 10 cm). The detailed exposure conditions for each mode are described in Table 1. The machine geometry for the MC simulations also followed the descriptions provided by the individual manufacturers.

**Table 1:** Exposure conditions of different modes in the CS9300 (Carestream Dental LLC, Atlanta, Georgia) and RAYSCAN α+ (Ray Co. Ltd, Hwaseong-si, Korea).

	CS9300		RAYSCAN α+	
	Facial	Dual jaw	Large jaw	Jaw
Field of view, cm	17 x 13.5	10 x 10	16 x 10	10 x 10
Tube voltage, kVp	90		80	
Tube current, mA	8	8	12	12
Exposure time, s	20	12	14	
Rotation angle, °	360			
Filtration, mmAl	2.8			
X-ray source to patient distance, cm	49.50		55.88	
Beam height (at rotation center), cm	13.5	10	10	
Beam width (at rotation center), cm	17	10	16	10

### 2. OSLD measurements

**Fig 1:**
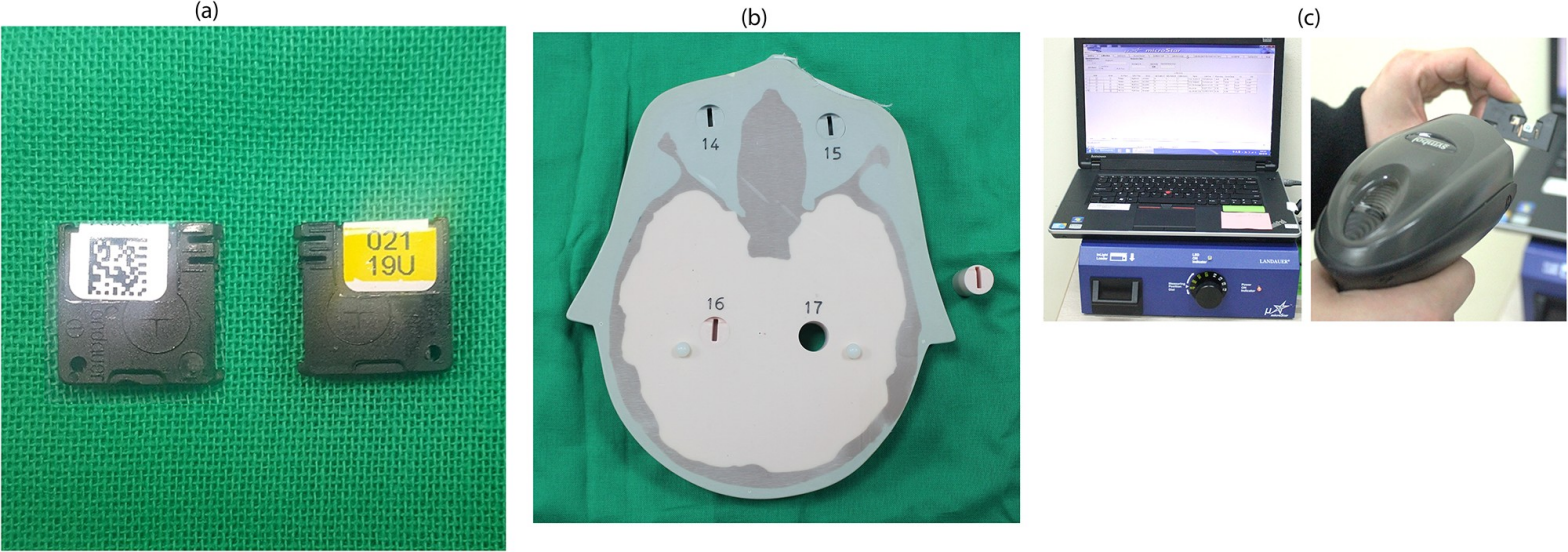
Experimental setting and facilitation for optically stimulated luminescence dosimeter (OSLD) measurements. (a) OSLD encased in a holder preventing light exposure. There is identification quick response (QR) code and identification number marked on the case. (b) Human tissue-equivalent phantom with dosimeter slots. (c) Dosimetry reader (MicroStar; Landauer) optimized for 80 kVp and a low dose (<30 mGy). Each dosimeter is identified with a QR code and can be read out.

An OSLD is a plastic disk containing aluminum oxide doped with carbon (Al_2_O_3_:C). This dosimeter absorbs radiation and this stored energy can be read out through light stimulation [12]. The dosimeter efficiently releases stored energy only when stimulated with light of 540 nm. Nonetheless, there is a potential risk that light of any wavelength may cause energy to be released from the dosimeter. To prevent this source of error, the dosimeter was encased in a plastic holder. Each holder case was tagged with a quick response (QR) code for the identification of the respective OSLD (Fig 1a).

**Fig 2:**
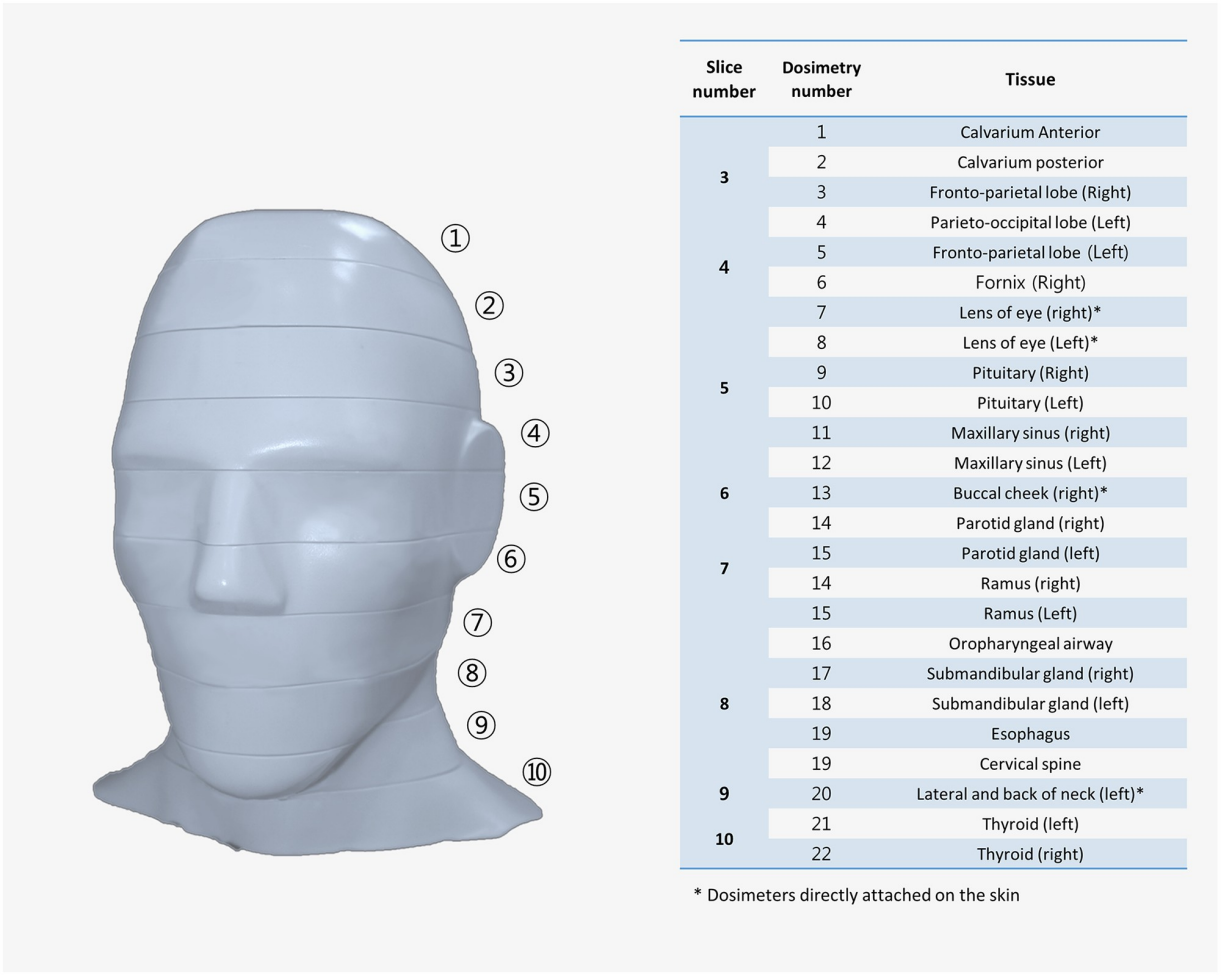
The location of optically stimulated luminescent dosimeters (OSLD) in an adult head and neck phantom (ATOM; CIRS, Norfolk, VA, USA) with the slice number of the phantom.

In total, 22 OSLDs (nano-Dot, Landauer, Inc., Glenwood, IL, USA) were placed in the head and neck organs of an adult head phantom (ATOM, CIRS, Norfolk, VA, USA). This phantom was composed of tissue-equivalent material (height, 173 cm; weight, 73 kg; physical density 1.6 g/cm^3^) and for each anatomical position, there was a slot for dosimeter placement (Fig 1b). Details of the OSLD locations and corresponding tissues are presented in Fig 2.

The phantom equipped with OSLD was exposed to four different examination modes (facial, dual jaw, large jaw and jaw) of two different units (CS9300 and RAYSCAN α+). All exposures were performed twice and the measured dose values were averaged for further calculations.

The reader (MicroStar; Landauer) was optimized for 80 kV and a low dose (<30 mGy), and each dosimeter was identified with QR code and read out (Fig 1c). The values were acquired as photon counts with an accuracy of approximate ±2%, which were converted to doses in units of mGy using the calibration curve based on the 80kV quality control (QC) set from manufacturer. Those values were then converted into organ doses, mostly following the method used by Ludlow et al. [8].

When multiple OSLDs were used for a single organ, the average value was used. For example, the mean values of the fronto-parietal lobe, parieto-occipital lobe, fornix, and pituitary were used to estimate the brain dose. The bone marrow dose was obtained considering the distribution of bone marrow in the mandible (0.8%), calvaria (7.7%), and cervical spine (3.8%) [13]. The bone surface dose was obtained by using a coefficient (the bone-to-muscle attenuation ratio) multiplied by the bone marrow value. The equation for the coefficient was as follows: -0.0618 × kV(p) × 2/3 + 6.9406 [14]. The irradiated proportion of the skin, lymph nodes, and muscles in the head and neck region was estimated to be 5%, and the irradiated proportion of the esophagus was estimated as 10%; these factors were taken into consideration in the organ dose calculation (Table 2) [15].

**Table 2:** Estimated fraction irradiated in tissues and tissue weighting factors recommended by the International Commission on Radiological Protection (ICRP).

	Fraction irradiated (%)	Tissue weighting factor	OSLD ID
Bone marrow	12.2	0.12	
Mandible	0.8		14, 15
Calvaria	7.7		1, 2
Cervical spine	3.8		19
Thyroid	100	0.04	21, 22
Esophagus	10	0.04	16
Skin	5	0.01	13, 20
Bone surface*	16.5	0.01	
Mandible	1.3		14, 15
Calvaria	11.8		1, 2
Cervical spine	3.4		21
Salivary glands	100	0.01	
Parotid	100		14, 15
Submandibular	100		17, 18
Brain	100	0.01	3, 4, 5, 6, 9, 10
Remainder tissue		0.12	
Lymphatic nodes	5		14, 15, 17, 18, 19
Muscle	5		14, 15, 17, 18, 19
Extrathoracic airways	100		14, 15, 17, 18, 16
Oral mucosa	100		14, 15, 16, 17, 18, 19
Eyes	100		7, 8

* Bone surface = bone marrow dose × bone/muscle mass energy absorption coefficient ratio (MEACR), MEACR = 0.0618 × 2/3 kVp + 6.9406 [14].

The organ doses were further integrated into the effective dose using the tissue weighting factors provided by the International Commission on Radiological Protection (ICRP) 2007 (Table 2) [8, 16]. The equation for the effective dose calculation is as follows: E = Σ W_T_ × H_T_, where E is the effective dose, W_T_ is the tissue weighting factor, and H_T_ is the radiation dose for specific organs [16].

### 3. Monte Carlo simulations

Monte Carlo (MC) simulations are a widely used technique in probabilistic analysis, in which random numbers are used to simulate the transport of radiation in a complex medium such as the human body [17]. When the physical information about an X-ray examination technique is given, the computer calculates the organ-absorbed dose through a MC simulation. In this study, commercial software commonly used for medical radiation dose calculation, PCXMC20Rotation (STUK, Helsinki, Finland) was used. This software includes the virtual phantom model height, (178 cm; weight, 73 kg; physical density 1.4 g/cm^3^) proposed by Cristy and Eckerman with modifications. This model uses simple three-dimensional shapes, such as balls, cuboids, and cylinders, to simulate the human body. According to the software manual, the following factors were set for the program to run: input dose, reference point, X-ray tube voltage, filtration, source-to-reference distance, and X-ray beam width and height at the reference point.

**Fig 3:**
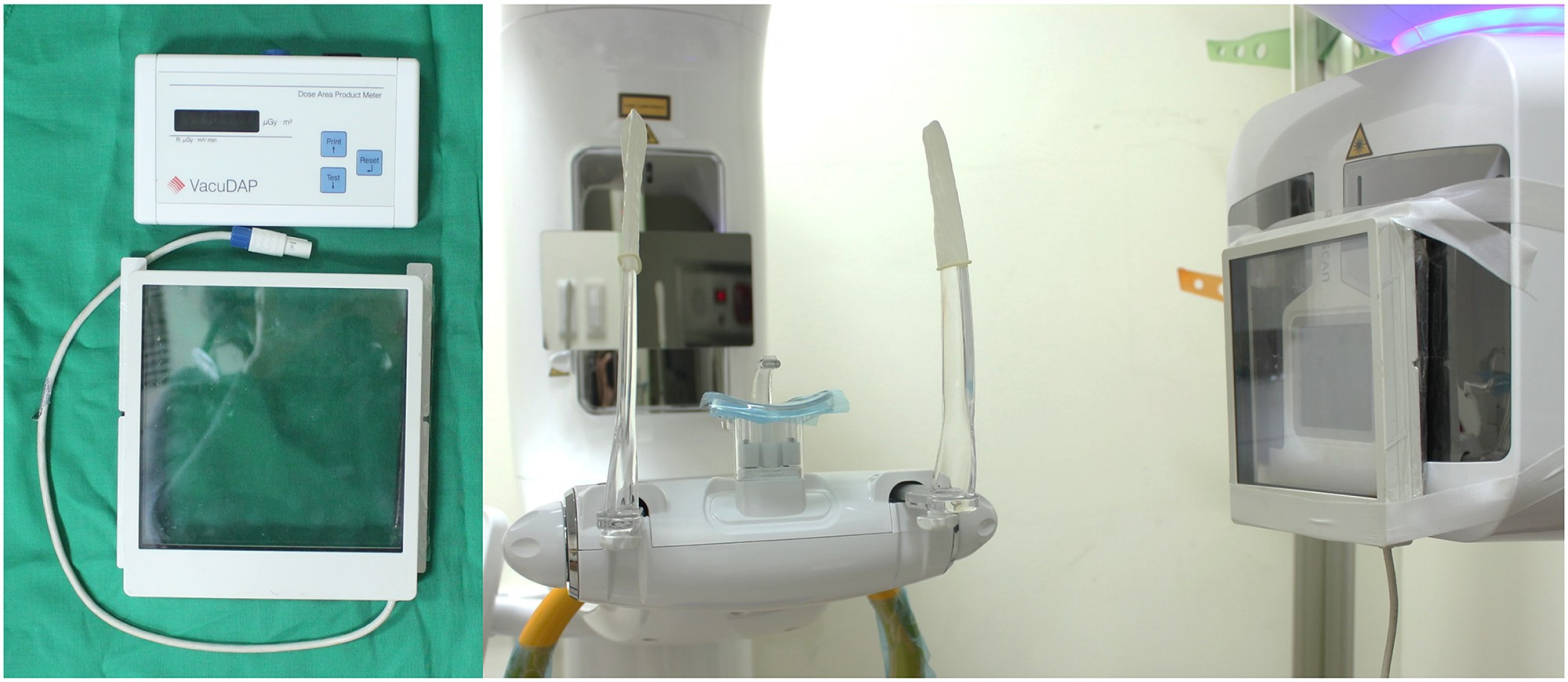
Dose-area-product (DAP) meter (VacuDAP™; VacuTec Meßtechnik GmbH, Dresden, Germany) for input dose measurement. An ion chamber was attached on the surface of the X-ray tube head for the measurements.

As an input dose, the exposure dose from the unit, expressed as the dose-area-product (DAP, mGy∙cm^2^), was selected and measured using a DAP meter (VacuDAP^™^; VacuTec Meßtechnik GmbH, Dresden, Germany). For each respective examination mode, measurements were performed twice and the mean values were used (Fig 3).

**Fig 4:**
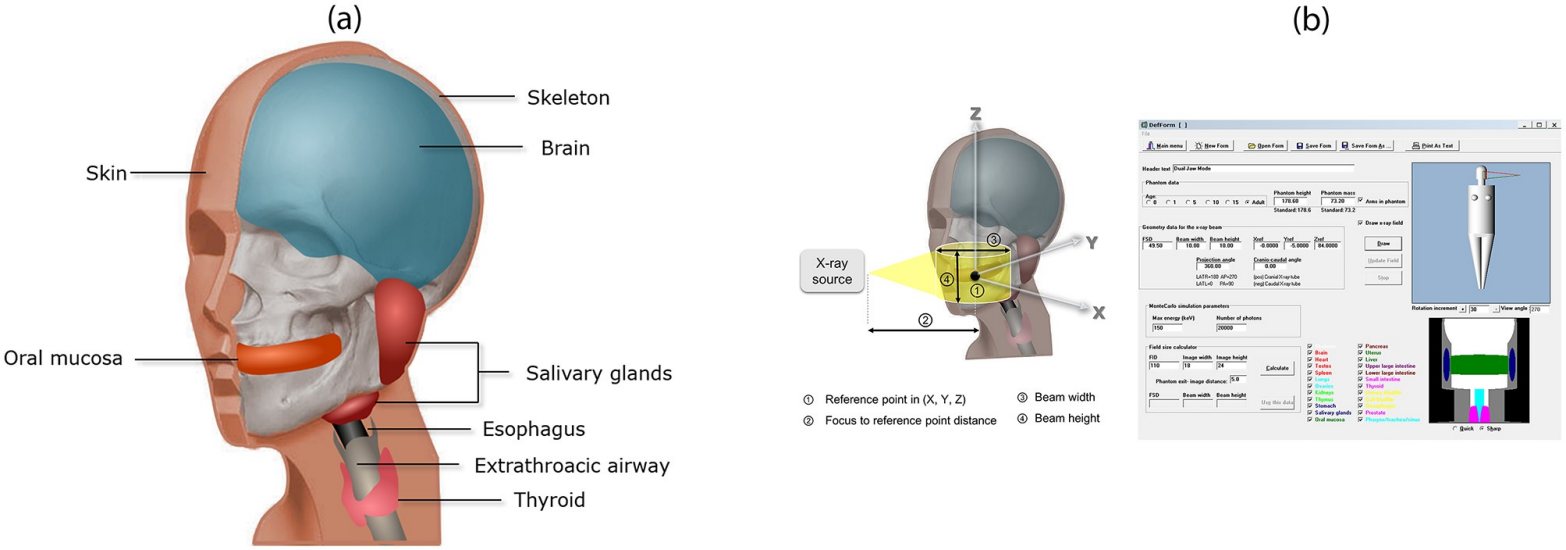
Virtual phantom and Monte Carlo (MC) simulation software. (a) Head and neck organs included in the virtual phantom. (b) Geometric variables required for the MC simulation and PCXMC20Rotation (STUK, Helsinki, Finland) software used in this study.

The reference point—the center of the X-ray unit during rotation, through which all X-ray beams pass—was determined by referring to the previous literature and marked as three-dimensional coordinates on the X-, Y-, and Z-axes (Fig 4) [5, 10]. In addition, the X-ray tube voltage, filtration, source-to-reference distance, and the beam width and height at the reference point were established according to the manufacturer’s specifications for each examination mode of the individual CBCT unit (Table 1).

## Results

The mean DAP values measured with the DAP meter were 215.1, 91.0, 176.6 and 167.9 mGy•cm2, respectively, for the facial and dual jaw modes of the CS9300 and the large jaw and jaw modes of RAYSCAN α+ (Table 3).

**Table 3:** Mean and standard deviation of dose-area-product (DAP) values measured with a DAP meter (mGy•cm^2^) for cone-beam computed tomography with different modes and devices.

CS9300		RAYSCAN α+	
Facial (17 × 13.5 cm)	Dual jaw (10 × 10 cm)	Large jaw (16 × 10 cm)	Jaw (10 × 10 cm)
215.1 ± 0.4	91.0 ± 0.4	176.6 ± 0.4	167.9 ± 0.6

**Fig 5:**
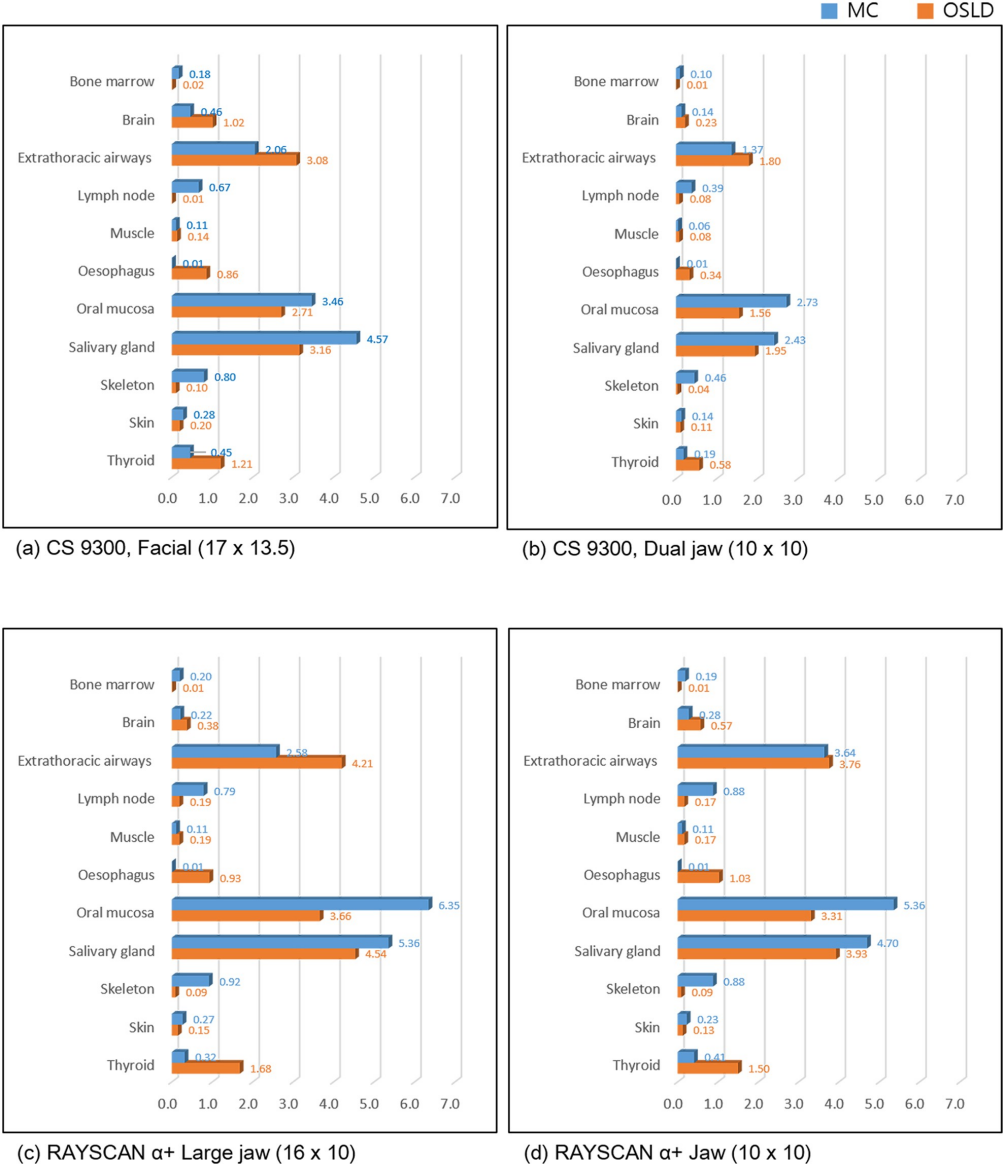
Organ dose of both methods according to the CBCT unit and examination mode. Note that the values varied according to each organ, while the overall trend was similar using both methods.

The organ dose varied according to the method, although the overall trend was similar in both methods (Fig 5). In other words, the organs found to have received relatively low doses using the OSLD method also mostly showed low doses using the MC method. Organs with high doses using the OSLD method also showed high doses using the MC method. In both methods, the oral mucosa and salivary glands were the two most-irradiated organs (Fig 5).

The OSLD-measured effective doses showed a tendency to have higher values than those obtained using the MC method. Only the dual jaw mode of the CS9300 device showed a higher effective dose using the MC method than using OSLD. The percent difference between the 2 methods was in the range of 4.0% to 14.3%. The dose difference between the methods decreased as the examination FOV decreased (Table 4).

**Table 4:** The effective dose obtained with the OSLD and MC methods, and the percent difference.

	Effective dose (μSv)			
	CS9300		RAYSCAN α+	
	Facial (17 × 13.5 cm)	Dual jaw (10 × 10 cm)	Large jaw (16 × 10 cm)	Jaw (10 × 10 cm)
OSLD method	181.4	90.7	228.5	213.8
MC method	160.9	94.4	198.0	195.2
	Percent difference (%)*			
	CS9300		RAYSCAN α+	
	Facial (17 × 13.5 cm)	Dual jaw (10 × 10 cm)	Large jaw (16 × 10 cm)	Jaw (10 × 10 cm)
	12.0	4.0	14.3	9.1

* Percent difference = $|\frac{effective\;dose\;(OSLD\;method)-effective\;dose\;(MC\;method)}{\frac{1}{2}\times effective\;dose\;(OSLD\;method)+effective\;dose\;(MC\;method)}|\times 100$

## Discussion

Since the development of CBCT in the dental field, its usage has grown rapidly, and research on the radiation dose of CBCT has always been of interest. Currently, various CBCT machines from numerous manufacturers are equipped with different exposure modes. In other words, the exposure dose and the patient-absorbed or effective dose vary across CBCT machines with different examination modes.

In 2015, Ludlow et al. meta-analyzed studies of the effective dose of CBCT conducted using dosimetry measurements. The values varied widely, from 46 to 1073 μSv for large FOVs and from 9 to 560 μSv for medium FOVs in each machine [3]. Assumed that the facial and large jaw modes investigated in this study corresponded to large FOVs and that the dual jaw and jaw modes had medium FOVs, the effective doses calculated in our study fell into these ranges, for both OSLD and the MC method. Differences in exposure conditions are likely to be the major contributors to the wide range of effective doses in different CBCT units with similar FOVs, although differences in the dose measurement method may also cause variation in overall effective dose assessment [3]. Thus, a consensus regarding the dose evaluation method is needed for a comparative analysis of effective dose reporting for each machine. Such standardization would be helpful for constructing a database of patient doses and establishing nationwide regulations for CBCT doses. Ludlow et al. studied the effective dose with OSLD and the same CBCT unit used in this study, CS9300, and reported effective doses of 204 and 76 μSv, respectively, for the facial and dual jaw modes [3]. Even in studies using the same methods and materials for dose evaluation, the effective dose values showed differences similar to, or even greater than, those between OSLD and the MC method. This discrepancy was probably caused by sampling error, as was also mentioned by Ludlow et al. [3]. Sampling error is defined as the influence of the location, distribution, and number of dosimeters used in each organ on the measured values. It is difficult to use the same number of dosimeters in every experiment performed by different experimenters for practical reasons, such as the cost of dosimetry. Phantom positioning within the CBCT unit during exposure is another challenge, as variation in positioning can cause large deviations in the resulting organ dose and effective dose. Sampling error might lead to an overestimation of the tissues distributed on large surfaces of the body, such as the oral mucosa, skeleton, and lymph nodes. Dosimeters cannot cover every site of those tissues.

The effective dose values obtained with the MC simulation showed relatively good agreement with those obtained using OSLD, with percent differences that were under 15%. Toivonen et al. [18] categorized the agreement as “good” when the difference between dosimetry and computer-simulated methods was below 25%. Second, user-dependent factors are limited throughout the entire measurement process using the MC method. According to previous studies on the use of the MC method for dose evaluation, information is needed on machine geometry, such as filtration, tube voltage, X-ray beam width and height, to conduct the simulation [10, 19, 20]. The manufacturers of the machines used in the current study provided the required information in the specifications. This method is also efficient in that it cost less than preparing a human tissue-equivalent phantom, dosimetry, and a dosimetry-reading device.

In order to apply the MC method correctly, it is important to use a standardized virtual phantom for the simulation [21]. In 2009, the ICRP introduced reference phantoms of female and male adults based on actual computed tomographic data from adult humans [22]. Only a few previous studies have used the ICRP reference phantoms, while others used computed tomographic scan data of the Rando-Alderson phantom [11, 23, 24].

In present study, the Cristy and Eckerman phantom contained in the software was used. This phantom model simulated human body organs using simplified three-dimensional shapes, such as cones, balls, and cylinders. Compared to the ICRP reference phantoms, it is not sophisticated enough to simulate precise estimations of the organ-absorbed and effective dose in dental CBCT. Highly precise results would have been achieved by using a more sophisticated human model for the MC method in this study.

The importance of fine aspects of the phantom model can also be inferred from the results of this study. The differences in the organ dose between OSLD and the MC simulation in this study were mainly due to differences in the tissue orientation and shape of the phantom model. Stratis et al. reported that when a phantom for the MC method in CT was used in a study of CBCT, the tissue-absorbed dose showed discrepancies because the head posture during CT examinations is different from that during CBCT examinations [24]. Similarly, in the present study, differences in the vertical location of radiation-sensitive organs, such as the thyroid gland, might have caused the observed discrepancies between the two methods [25]. In 2015, Ludlow et al. [3] argued that DAP is not appropriate to be used for obtaining the effective dose. This statement is true, if we simply convert DAP values into the effective dose using a conversion coefficient. Several studies have attempted to find a conversion coefficient to obtain the effective dose of CBCT, but coefficients vary across individual CBCT models with unique geometries [26, 27]. In contrast, MC simulations calculate the effective dose taking individual machine geometry into consideration. Thus, the MC method may produce more precise results based on DAP values than are possible by simply converting DAP values into an effective dose.

In the present study, DAP measurements were performed using a DAP meter. The DAP measurement procedure is not experimenter-specific; however, it requires equipment including an ion chamber, DAP meter, and cables. The procedure also takes time and is laborious for the experimenter. Fortunately, recent CBCT machines provide DAP values according to the exposure conditions. Although these values are not measured in real time—instead, they are predetermined by the manufacturer—MC simulation software with a precise reference phantom may make it possible to obtain an approximate effective dose that does not depend on the experimenter or the measurement method.

In conclusion, effective doses for various CBCT models and examination modes have been frequently reported, and extensive data have been gathered [3, 28]. To contribute further to the accumulation of big data on this topic, the effective dose obtained by two different methods and CBCT machines was reported in this study. The ultimate goal of effective dose assessment and data accumulation is dose reduction and regulation for the patients’ benefit. To achieve this goal, a consensus regarding the dose evaluation method is essential. In addition, development of a relatively accurate and easy-to-use method would contribute to the acquisition of more dose data. Therefore, we cautiously suggest the use of MC simulations based on a reference phantom for dose evaluations in the future.

## Supporting information

S1 Data (XLSX)
